# Dr. Lorenzo N. Phinney

**Published:** 1915-06

**Authors:** 


**Dr. Lorenzo N. Phinney,** Geneva 1866, died at his home in Wappinger's Falls, N. Y., April 20, aged 74. He was surgeon of the 193d N. Y. Infantry.

